# Third-Generation Anti-CD47-Specific CAR-T Cells Effectively Kill Cancer Cells and Reduce the Genes Expression in Lung Cancer Cell Metastasis

**DOI:** 10.1155/2021/5575260

**Published:** 2021-06-02

**Authors:** Huyen Thi La, Dao Bich Thi Tran, Hai Manh Tran, Linh Trong Nguyen

**Affiliations:** ^1^Institute of Biotechnology, Vietnam Academy of Science and Technology, Hanoi, Vietnam; ^2^Graduate University of Sciences and Technology, Vietnam Academy of Science and Technology, Hanoi, Vietnam

## Abstract

CD47 is a cell surface glycoprotein molecule, belonging to the immunoglobulin superfamily, binding to various proteins including integrins, thrombospondin-1, and signal regulatory protein *α* (SIRP*α*). CD47 is an important tumor antigen for the development and progression of various cancers. This study designed the chimeric antigen receptor T-cell (CAR-T) to bind to the CD47 to inhibit the expression of CD47. We used the complementarity-determining regions (CDRs) of the B6H12 mouse antibody grafted onto the IgG1 framework to create the humanized single-chain variable fragment (scFv) with linker (G4S)x3. scFv was used to design the chimeric antigen receptor with the structure CD8signal-CD47scFv-CD8a hinge-CD4TM-CD28-41BB-CD3*ζ*, which was then transformed into T lymphocytes by the lentivirus to create third generation of CAR-T. Results revealed that the new CAR-T cells efficiently killed A549 cancer cells. CAR-T inhibited the expression of genes involved in metastasis and invasion of cells A549 including beta actin, calreticulin, and cyclooxygenase 2 at mRNA levels.

## 1. Introduction

Current experimental evidence suggests an important role of immunoglobulins in the formation of cancerous tumors. CD47 is one of the common cell surface immunoglobulin-like glycoproteins found in leukemia or other cancers such as ovarian cancer, pancreatic cancer, glioma, and others [1–6]. In addition to functionally interacting with proteins such as integrins and thrombospondin-1, CD47 is considered a macrophage immune checkpoint when interacting with signal regulatory protein *α* (SIRP*α*) [7]. This activates SIRP*α*, phosphorylates the immunoreceptor tyrosine-based inhibition motif (ITIM), recruits SHP-1 phosphatases to the cell membrane, and then limits accumulating myosin at the surface of the cell, which in turn prevent phagocytosis. The expression of CD47 is related to tumor growth and is proposed to be a critical clinical prognostic factor and mortality [6, 8]. The presence of CD47 has been found in patients with metastasis, immune evasion, or cell migration [8, 9].

Previous literature found the success in the use of animal and humanized antibodies for blocking the CD47 pathway, resulting in the suppression and block of tumor growth [5, 10]. In which, the use of chimeric antigen receptor T-cell (CAR-T) therapy is premise to against leukemia and solid cancers. A previous study revealed that anti-CD47 CAR-T was effective in pancreatic cancer cells [11]. The CAR structure consists of an extracellular antigen-binding domain called scFv which fuses with a hinge domain, transmembrane domain, extracellular spacer, intracellular signaling domain, which is responsible for activating T-cell, costimulatory domain (CD28, 4-1BB, CD27, or other domains), and CD3*ζ*-activated domains [12]. The humanized CAR structure is potential to extend the durability of CAR-T cells, which can enhance the duration of antimicrobial responsive activity of CAR-T cells to pathogenic cells. Therefore, it is important to choose a human-nature scFv structure.

Selection of appropriate hinge domain is also important, which is attributable to the connection and affinity of bonding positions. The hinge domain has previously been neglected, but preclinical experiments have demonstrated that the hinge region plays an important role in the regulation of binding affinity and signaling [13]. Literatures revealed that the hinge domain of CD8*α* was more effective than that of CD28 given that it could release lower cytokine concentration and cell death [14]. Similarly, selecting the transmembrane domain is vital when it plays a role in connecting the hinge domain and the intracellular domain. Using the transmembrane domain of CD8-*α* showed lower levels of T cell activation-induced death compared to that of CD28 [14].

Efforts to improve CAR-T cells by changing intracellular domain have been implemented recently. In literature, 4-1BB transmembrane performed better than CD28 transmembrane in clinical and laboratory settings, because 4-1BB costimulation could reach a higher level and longer duration than that with CD28 costimulation [15, 16]. However, the limitation of CAR-T therapies cannot be resolved with a single costimulation domain. Thus, the third generation of CAR-T cells is designed with two costimulation domains. Preclinical trials showed that this strategy increased endurance, proliferative, and antitumor activities [17–19]. In this study, we designed the structure CD47scFv-hinge-CD4TM-CD28-41BB-CD3*ζ* to create CAR-T cells to destroy CD47+cancer cells, more specifically, lung cancer cell line A549. A549 is the nonsmall cell lung cancer cell line (NSCLC), which accounts for 85% of all lung cancers. A549 exhibited high CD47 expression and was used in the CD47-related metastasis study [20]. In this study, we tested the toxicity level, determined cytokine content, and ability to regulate cell metastasis through decreased activity of related genes.

## 2. Materials and Methods

### 2.1. Materials Preparation

#### 2.1.1. Cell Lines

The HEK293FT (Invitrogen) and A549 cell lines were supplied by the Inserm U 853 Laboratory, French Institute of Health and Medical Research. Human peripheral blood mononuclear (PBMC) cells were isolated from the donor's whole blood.

#### 2.1.2. Chemical and Biological Products

Chemical and biological products include the following:

- The plasmids: pcDNA 3.1+CAR gene (genscript), Vector pFUGW (from addgen, code 14883), pCMV-dR8.2dvpr (from addgen, code 8455), and pCMV-VSV-G (from addgen, code 8454)
- Cell culture medium DMEM, FBS, Opti-MEM (Gibco); penicillin/streptomycin, Trypsin, Trypan blue (Pan Biotech), Ficoll-PaqueTM (GE Healthcare)
- T-CD3 cell separator kit; TexMAXS Medium, IL-2; T Cell TransAct were obtained from Miltenyi Biotec
- Human IFN gamma ELISA Kit; Human IL-2 ELISA Kit (Abcam)
- The enzyme T4 DNA ligase; EcoRI and BamHI (BioLabs); DNA marker (Thermo); Plasmid DNA MIDI KIT (QIAGEN); Gel Extraction Kit (QIAGEN); Lipofectamine™ 3000 Transfection Reagent, P3000™ Reagent (Invitrogen).
- Easy-BLUE™ Total RNA Extraction Kit (iNtRON), real-time Luna universal one-step RT-qPCR kit (Biolabs); Total DNA extraction kit (QIAgen); PCR master mix (Promega)
- Gene test primer sequences are presented in Table 1

### 2.2. CAR Structure Design

The CDR from the mouse antibody B6H12.2 were grafted onto the VH and VL of IgG1 framework to create the humanized scFv generator with the 3xGGGGS linker. The CAR gene structure included the CD8a lead sequence, a part of the extracellular domain of CD8a and the CD4, and CD28 transmembrane domains; intracellular domain 4-1BB and the CD3zeta. The structure was placed on genescript synthesis and gene sequence optimization. The CAR structure is attached to the vector lentivirus pFUGW (addgene) through the two enzymes BamHI and EcoRI. CAR structure was cut with two enzymes BamHI and EcoRI and transferred to vector pFUGW, selected clone by transformation into E. coli and colony PCR, and separated recombinant plasmid by DNA midi kit (QIAgen). Plasmids and their concentration were examined by using the agarose gel electrophoresis and nanodrop measurement, respectively.

### 2.3. Prepare the Lentiviruses

CAR lentiviruses were created by using HEK 293FT cells (Invitrogen) according to the instructions of the University of Texas, MD Anderson Cancer Center with some modifications [26]. 3.5 × 10^6^ HEK cells were grown on a 6-well plate (10 cm^2^) in antibiotic-free DMEM medium; and cells covered 90% of the plate surface after one day. Then, a 10 *μ*g three vectors were simultaneously transferred including FUGW/CAR, pCMV-dR8.2dvpr, and pCMV-VSV-G in a 5 : 5 : 1 ratio into HEK 293FT cells. The medium volume in the plate reached 0.5 mL opti-MEM. With the support of Lipofectamine 3000 Transfection Reagent and P3000 Reagent, according to the manufacturer's manual [27], the cell plate was incubated at 37°C, 5% CO2 for approximately six hours. Then, the transfection was removed, and 3 mL of the DMEM medium with 10% FBS and 1% P/S antibiotic was added for each well. The cell plates were incubated at 37°C, 5% CO2, and the lentivirus solution was collected after 72 hours. This solution was filtered with a 0.45 *μ*m filter; then, it was concentrated five times with a Microsep™ Advance column. Virus titration was determined by quantitative RT-PCR using Biolabs reagent on the QIAgen Real-time machine.

### 2.4. Determining the Number of Lentiviruses by Real-Time PCR Method

A 100 *μ*L lentivirus was used to extract and obtain 40 *μ*L RNA by using trizol easy-BLUE™ Total RNA Extraction Kit (iNtRON). DNA was removed by RcoRI and DnaseI (Biolabs), and 1.55 *μ*L RNA was used as a template for the Real-time PCR reaction with Sybr green and TestCar primer pair. Following procedures were set up: reverse transcription: 55°C for 10 minutes; initial denaturation: 95°C for 1 minute then run 45 cycles with denaturation 95°C for 10 seconds, 60°C for 30 seconds; and melting curve analysis with temperature range 60-95°C. The calibration curve was built from pcDNA3.1 carrying the CAR gene with the same primer pair as above.

### 2.5. T-Lymphocyte Separation Procedure

The T lymphocyte separation procedure was carried out according to the Miltenyi's protocol [28, 29]. Specifically, 10 mL of peripheral blood was diluted in 20 mL of PBS 1x (Invitrogen) then added to 15 mL of Ficoll (Pan Tech). It was centrifuged with 3000 rpm for 30 minutes in order to obtain the single-cell layer. Mononuclear cells were washed by using 5 mL of PBS 1x and collected cells after centrifuging 200 rpm for 15 minutes at 20°C.

The obtained PBMCs were mixed in PBS 1x at a concentration of 10^7^ cells/100 *μ*L. The microbead CD8 and CD4 were mixed in 1 : 1 ratio (100 *μ*L microbead CD8+100 *μ*L microbead CD4, with 20*μ*L for 10^7^ cells). The mixed solution of microbead CD8 and CD4 was gradually supplemented to cells. Then, the sample was incubated at 4°C for 15 minutes. Cells were washed with PBS 1X+BSA 0.5%+EDTA 2 mM buffer (10^7^ cells needed 1-2 mL of buffer, or 7 mL for 5.25 × 10^7^ cells). The tube was centrifuged at 300 rpm for 10 minutes; then, cell sediment was collected. Cells were dissolved in 500 *μ*L of PBS 1X buffer+BSA 0.5%+EDTA 2 mM.

The MS column was attached to the magnetic machine. This column was washed twice with 500 *μ*L of PBS 1X buffer+BSA 0.5%+EDTA 2 mM. The fluid flow was let completely through the column. Then, the entire cell was put onto the column and let the fluid run through the column. The column was washed three times with 500 *μ*L of PBS 1X buffer+BSA 0.5%+EDTA 2 mM, and the negative fragment was collected in a separate tube. Then, the column was removed from the machine and added 1 mL of PBS 1X+BSA 0.5%+EDTA 2 mM buffer to blow cells out of the column. The piston was used and attached to the column to completely push the cells out of the column. Positive fragment was obtained in a separate tube. The extracted T cells were then counted and centrifuged 1000 rpm for 10 minutes to collect cells. Cells were dissolved in culture medium and add T Cell TransAct at the rate (100 *μ*L/10^7^ cells). Cells were cultured at 37°C, 5% CO2.

### 2.6. Cell Culture and Transfer

The cell was stirred with a pipette and sucked all the cytosol into the falcon tube and centrifuged 1000 rpm for 5 minutes to collect the cells. Cells were dissolved in TexMACS medium and supplemented with 300 IU/mL IL2 and T-cell TransAct at a ratio of 100 *μ*L/10^7^ cells. Cells were cultured at 37°C, CO2 5%.

### 2.7. Infecting Lentiviruses into T-Lymphocytes

The procedure of infecting lentivirus into T-cells was carried out according to the instructions of Genemedi company [30], with an infectious multiple (MOI) of 50 in opti-MEM medium, and the centrifugation of 200 g for 1 hour using the Sorvall legend RT centrifuge. On the following day, the infecting medium was completely removed and the cells were reconstituted in TexMAXS, 300 U/mL IL-2 medium. After 3-5 days, a part of the cells was collected, replaced the new TexMAXS medium with 300 U/mL IL-2, and transacted to maintain the cell density at 1 × 10^6^ cells/mL.

### 2.8. Flow Cytometry

The BD MultitestTM CD3/CD8/CD45/CD4 kit (Becton, Dickinson, USA) was used according to manufacturer's instructions [31]. First, a pipette was used to suck 20 *μ*L BD Multitest CD3/CD8/CD45/CD4 into the bottom of the tube. A 50 *μ*L of cultured T cells and CAR-T cells were transferred to the bottom of the tube and mixed them. The tube was capped and swirled gently for mixing. Tubes were incubated in the dark for 15 minutes at room temperature (20-25°C); then, 450 *μ*L of 1X BD FACS sediment was added to the tube. The tube was capped and swirled gently for mixing. Next, the tube was incubated in the dark for 15 minutes at room temperature (20°C-25°C). Cells were analyzed by using the BD FACS Canto II equipment.

### 2.9. Determining the Gene Transfer Efficiency by Relative Quantitative PCR

The T lymphocytes and CAR-T cells were used to extract the total DNA using the QIAgen extraction kit. Then, the Real-time PCR was performed with Real-time Master Mix Luna universal RT-qPCR kit (Biolabs) between CAR gene and GADPH gene. A 1.55 *μ*L RNA was used as a template for Real-time PCR reaction with Sybr green and TestCar primers. The program was set up for the Real-time PCR machine as follows: reverse transcription: 55°C for 10 minutes; initial denaturation: 95°C for 1 minute then run 45 cycles with denaturation 95°C for 10 seconds, 60°C for 30 seconds; and melting curve analysis with temperature range 60-95°C. By using Livak's 2-DDCt method, transformation efficiency was calculated by examining the presence of the CAR gene segment in the target cell genome, compared with the reference gene GAPDH. The calculation formula is as follows:

(1) $$\begin{array}{c} \text{RQ}=\frac{2^{∆\text{Ct}\left(\text{target}\right)}}{2^{∆\text{Ct}\left(\text{reference}\right)}}=2^{\left[∆\text{Ct}\left(\text{target}\right)-∆\text{Ct}\left(\text{reference}\right)\right]}=2^{\left[\text{Ct}\left(\text{target},\text{calibrator}\right)-\text{Ct}\left(\text{target},\text{test}\right)\right]-\left[\text{Ct}\left(\text{ref},\text{calibrator}\right)-\text{Ct}\left(\text{ref},\text{test}\right)\right],} \end{array}$$

### 2.10. Determining Gene Expression Involved in A549 Cell Metastasis

A549 cells were cultured in DMEM medium supplemented with FBS and the antibiotic P/S. The cells were then divided into 24-well plates (Nunc) with 5 × 10^4^ cells per well and cultured overnight. In the following day, the medium was removed and replaced with 5 × 10^4^ T cells and CAR-T in TexMAXS medium containing 300 U/mL IL-2 and T-cell TransAct™. This process repeated three times. After 24 hours, CAR-T and T cells were completely removed from the culture medium and washed again with PBS 1x. The cell A549 was obtained by trypsinization, and RNA was extracted with trizol. Then, the expression of beta actin, carl, and COX-2 genes was analyzed with primer pairs listed in the material through the Real-time PCR method with GAPDH gene as reference. The expression efficiency of each gene in cell A549 in different samples was calculated by the formula:

(2) $$\begin{array}{c} \text{RQ}=\frac{2^{∆\text{Ct}\left(\text{target}\right)}}{2^{∆\text{Ct}\left(\text{reference}\right)}}=2^{\left[∆\text{Ct}\left(\text{target}\right)-∆\text{Ct}\left(\text{reference}\right)\right]}=2^{\left[\text{Ct}\left(\text{target},\text{calibrator}\right)-\text{Ct}\left(\text{target},\text{test}\right)\right]-\left[\text{Ct}\left(\text{ref},\text{calibrator}\right)-Ct\left(ref,test\right)\right],} \end{array}$$

- Ct (T/Tg) was the cycle threshold of the target gene (Tg) in the sample (T)
- Ct (T/Ref) was the cycle threshold of the reference gene (Ref) in the sample (T)
- Ct (C/Tg) was the cycle threshold of the target gene (Tg) in the control (C)
- Ct (C/Ref) was the cycle threshold of the reference gene (Ref) in the control (C)

Based on these parameters, the cycle threshold of the target gene on the sample (T) and control (C) was normalized by calculating the difference of the DCt of the target gene from the reference gene:

Sample: DCt (T) = Ct (T/Tg) − Ct (T/Ref)

Control: DCt (C) = Ct (C/Tg) − Ct (C/Ref)

The DCt of the sample was normalized by calculating the difference between DCt (T) and DCt (C): DDCt = DCt (T) − DCt (C). From there, the expression rate of the target gene on the sample compared to the control was calculated.

### 2.11. Cell Toxicity Test Using MTT

The cell toxicity test was performed with the procedure suggested by Merck KGaA company [32]. The target A549 cells were divided to 96-well plates (Nunc) with 1 × 10^4^ cells per well and were cultured overnight. In the following day, the medium was removed and replaced with 1 × 10^4^ T or CAR-T cells cultured in the TexMAXS medium containing 300 U/mL IL-2 and T-cell TransAct™. This process was repeated three times. Cells were monitored for an additional 2 days. The survival/death rate was determined by MTT (3-(4,5-Dimethyl-2-thiazolyl)-2.5 diphenyltetrazolium bromide-sigma). The obtained data was then analyzed using the Excel software to determine the cell viability (% Cell viability).

(3) $$\begin{array}{c} \%\text{Cell viability}=\frac{\left(\text{OD}490\text{ analytical sample}-\text{OD}490\text{ blank}\right)}{\text{OD}490\text{ control sample}-\text{OD}490\text{ blank}}*100\%, \end{array}$$

Control sample: the sample had only cells and cell culture medium.

Analytical sample: reagent sample had corresponding concentrations in cell culture medium.

Blank: cell culture medium.

### 2.12. Cytokine Test by ELISA

The target A549 cells were cultured on 48-well plates with a number of 5 × 10^4^ cells per well, overnight at 37°C, 5% CO2 in DMEM F12 medium containing 10% FBS, 1% P/S. After 24 hours, the culture medium was completely removed and added 5 × 10^4^ effector cells (CAR-T cells or nontransgenic T cells) into wells containing A549 cells with 200 *μ*L TexMAXS. This process was repeated three times. After 24 hours, 150 *μ*L of the medium was transferred to a centrifuge tube and centrifuged at 1000 rpm for 5 minutes to remove the remaining cells. The supernatant was used for cytokine analysis (IFN gamma, IL-2) as per Abcam's cytokine KIT (Human IL-2 ELISA Kit; Human IFN gamma ELISA Kit).

### 2.13. Statistical Analysis

Data were analyzed and compared between 2 groups, T-cell and CAR-T, by Student *T*-test. The statistically significant difference was determined with *p* value < 0.05.

## 3. Results

### 3.1. Design of Humanized CD47 scFv from Mouse Antibody B6H12

**Figure 1:**
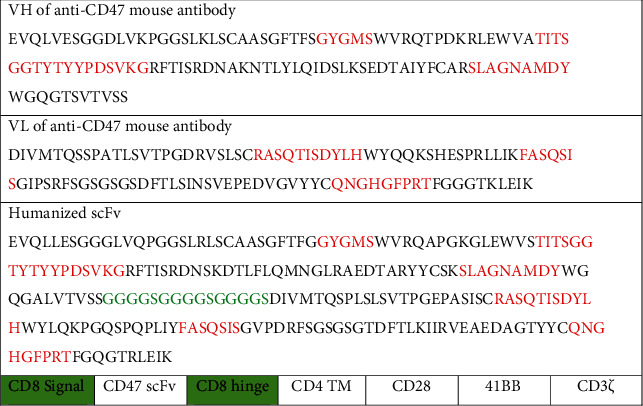
Amino acid sequence VH, VL of B6H12 mouse antibody; anti-CD47 humanized scFv antibody; and structure of chimeric antigen receptor.

CDR domains of the B6H12 mouse antibody sequence were determined according to the Kabat theory. The CDR domains of the mouse antibody were grafted to the human VH and VL IgG1 framework, using linker (G4S)x3 to create humanized scFv antibodies (Figure 1). Humanized anti-CD47 scFv was attached to phagemid pHEN2; then, the recombinant phagemid was transferred to E. coli TG1 strain, using helper phage to form M13 phage carrying CD47 scFv attached to the phage's gIII. The CD47 scFv phage was tested by ELISA for binding to recombinant CD47 antigens and A549 cell-derived antigens. Results show that humanized CD47 scFv had a high affinity for CD47.

### 3.2. Design of Chimeric Antigen Receptor (CAR) Carrying Anti-CD47 Humanized scFv and Produces Lentiviruses

**Figure 2:**
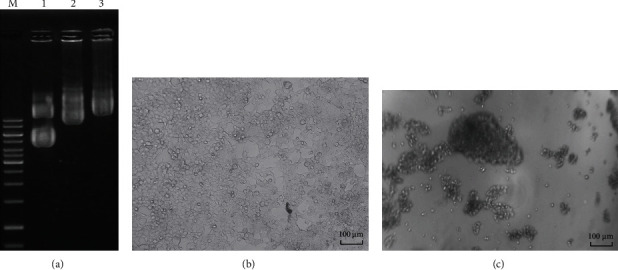
(a) Agarose electrophoresis of pCMV-VSV-G plasmids (runway 1); FUGW carried the CAR gene (CD47 scFv-hinge-CD4TM-CD28-41BB-CD3*ζ*) (runway 2); pCMV-dR8.2 dvpr (runway 3). (b) HEK cells in control sample. (c) Broken HEK cells produce lentivirus.

Humanized anti-CD47 scFv was used to design the structure of CD8signal-scFv anti-CD47-hinge-CD4TM-CD28-41BB-CD3*ζ* receptor (Figure 1). The conduction region was the conduction region of CD8a (21aa), followed by the humanized scFv (240aa), the hinge domain of CD8a (119aa), the transmembrane domain CD4 (22aa), the CD28 costimulation domain (41aa), and 41BB (42aa) and CD3*ζ* (113aa) activation domain. This structure was used to infer the DNA sequence and was optimized for the human cell expression by a software via increasing the CAI value from 0.80 to 0.95. Two ends of the CAR gene attached to the two cutting positions of the EcoRI and BamHI enzymes. The CAR gene structure was synthesized by Genscript (USA) which is attached to the vector pcDNA3.1. The gene coding for CAR was cut with the enzyme EcoRI and BamHI and attached to the vector pFUGW (14883). pFUGW/CAR (CD47scFv-hinge-CD4TM-CD28-41BB-CD3*ζ*) together with shell vectors pCMV-dR8.2dvpr (8455) and pCMV-VSV-G (8454) were extracted using midiprep plasmid extraction kit (QIAgen). These plasmids were then used to generate lentivirus through the HEK 293FT cell (Figure 2).

The lentivirus was quantified by the Real-time PCR method with standard curves constructed from vector pFUGW/CAR (CD47scFv-hinge-CD4TM-CD28-41BB-CD3*ζ*) and TestCarF/R primers. The amount of lentivirus was 7.5 × 10^7^ cfu/mL.

### 3.3. Separation of T Lymphocytes and Creation of CAR-T Cells

**Figure 3:**
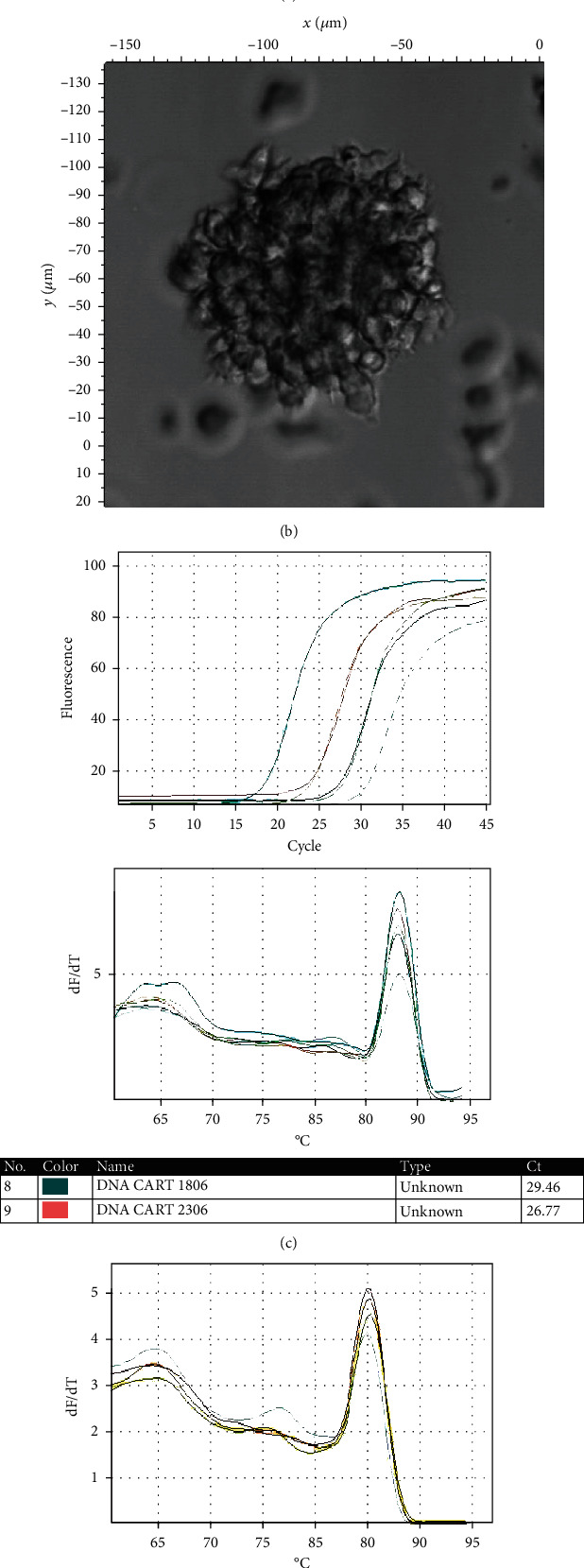
(a, b) The mixture of CAR-T cells was taken on confocal microscope C1si (Nikon) with different magnifications. (c) Results of Real-time DNA analysis of CAR-T cells with TestCarF/R primers. (d) Real-time DNA analysis of CAR-T cells with GADPHF/R primers.

**Figure 4:**
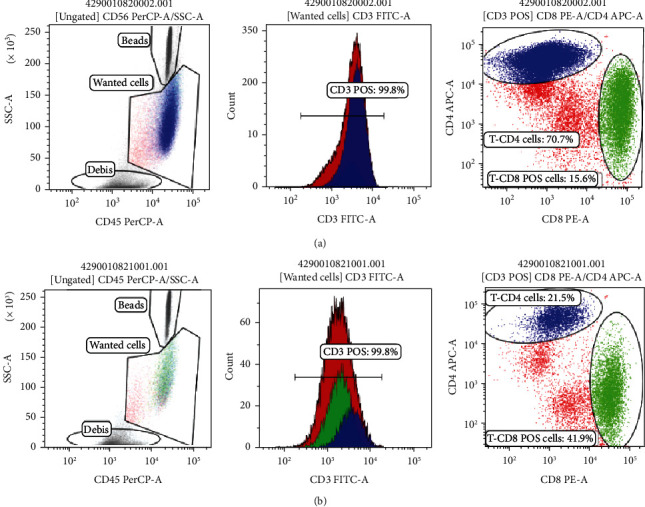
Flow cytometry results of transgenic–CAR-T cells (a) and T-lymphocytes (nontransgenic) after 14 days of culture (b).

A 10 mL volume of peripheral blood was used for mononuclear cleavage using ficoll (Pan Tech) and Milltenyi's CD4 and CD8 T-lymphocyte separation kit. The T lymphocytes obtained in the TexMAXS culture medium were fused with 300 U/mL IL-2; supplementing T Cell TransAct with the ratio 100 *μ*L/10^7^ cells. Cells were cultured at 37°C, 5% CO2. Lentivirus containing the structure CD47scFv-hinge-CD4TM-CD28-41BB-CD3*ζ* was infected to T lymphocytes with MOI = 50 in OptiMEM medium. After 24 hours, the old medium was removed and replaced the new medium TexMAXS, 300 U/mL IL-2, and 1% T-cell TransAct. The effect of creating CAR-T was evaluated by the Real-time PCR method to quantify CAR gene and flow cytometry. The results showed that after 18 days of culture, the number of cells carrying DNA encoding CD47scFv-hinge-CD4TM-CD28-41BB-CD3*ζ* reached 70-85% (Figure 3) and over 25% of CAR-expressing cells (Figure 4). In CAR-T cells therapy, after the gene transfer process, a mixture of T cells and CAR-T cells was used in therapy without cleavage. Therefore, the transgenic cell mixture would be used for follow-up studies with nontransgenic T-cells as a control.

### 3.4. Capability of CAR-T Cell to Release Cytokines

**Figure 5:**
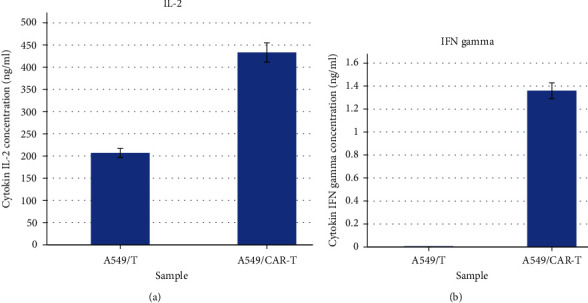
(a) IL-2 and (b) IFN gamma secretion of CAR-T and T cell when interacting with cancer cell lines A549.

By using the ELISA cytokine (Abcam) kit, results of CAR-T cells' ability to release cytokines IL-2 and IFNg showed that CAR-T cells were capable of releasing these cytokines when interacting with cancer cells A549. Specifically, interactions between CAR-T cells and A549 cancer cells produced 1.36 ng/mL IFNg; meanwhile, the interaction of T cells with cancer cells A549 did not produce any IFNg. For IL-2, T cells when interacting with cancer cells A549 could secret 206.8 ng/mL IL-2, which was lower than the amount of IL-2 secreted when CAR-T cells interacted with A549 cells (433.2 ng/mL). The differences were statistically significant with *p* < 0.05 (Figure 5).

### 3.5. Evaluating the Effect of Killing Cancer Cells A549

**Figure 6:**
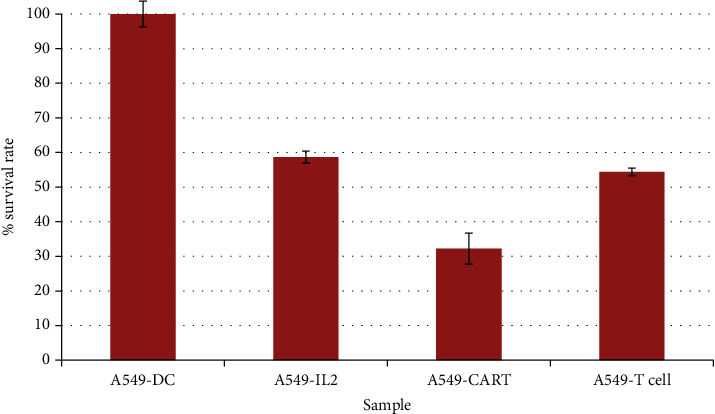
Results of T-cell and CAR-T cell toxicity test on A549 cells.

Results showed that CAR-T cells killed A549 cells over 60%, which was better than that of T cells (54.41%). The difference was statistically significant with *p* < 0.05 (Figure 6). Theoretically, CD47 CAR-T cells kill CD47+cancer cells. In the study, our results also showed that CD47 CAR-T cells were toxic to the A549 cell line in the MTT assay.

### 3.6. Evaluating the Inhibitory Effects on the Expression of Genes Involved in Cancer Metastasis on Cell Line A549

**Figure 7:**
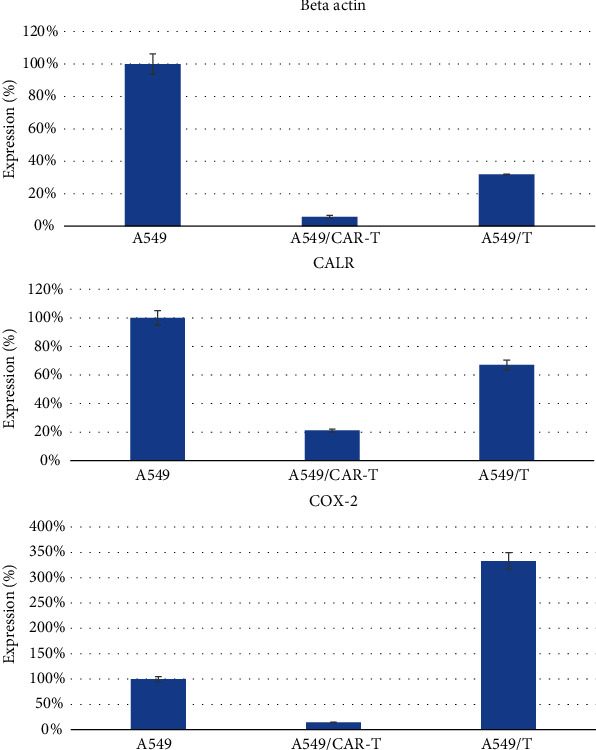
Comparison of expression of genes involved in metastasis in A549 cells before and after exposure to CAR-T cells and T cells.

Analysis of the expression of genes by the Real-time PCR showed that, after exposure to anti-CD47 CAR-T cells, A549 cells decreased the expression of beta actin, calreticulin, and cyclooxygenase 2 (COX-2) genes when compared to A549 cells in the control group and the T-lymphocyte exposure group. Specifically, after exposure to CAR-T cells and T cells, the A549 cells expressed beta actin gene by only 6% and 32% compared with that in the control group, respectively. For the calreticulin gene, after exposure to CD47 CAR-T cells and T cells, A549 cells expressed this gene by only 21% and 67% of the control, respectively. Particularly for the COX-2 gene, T lymphocytes reactivated the expression of COX-2 genes in cell A549 (expression increased 333% compared to the control group with *p* < 0.01); meanwhile, CAR-T cells decreased COX-2 expression by 15% compared to the control (Figure 7).

## 4. Discussion

In this study, humanized CD47-CAR-T cells showed a potential in killing the A549 cancer cell lines. This is because A549 cells are one of the CD47-overexpressing cancer cell lines [20]. Although CD47-CAR-T exhibits effective destruction of A549 cells, the amount of cytokine secreted after exposure to the target cells was not high. This is probably due to the structure of CD47scFv-hinge-CD4TM-CD28-41BB-CD3*ζ*. This structure has the CD8*α* as the hinge domain, which has been shown in previous studies that this hinge domain released lower cytokine concentrations (e.g., IFNg and TNF), reducing the activation-induced cell death and being less susceptible to AICD than cells with CD28-derived domains [14]. We selected cytokines IL-2 and IFNg after referring to previous studies [33], which identified IL-2, TNF, and IFNg as CAR-T's cytokines. Indeed, the cytokine storm appeared to be the result of nonspecific T-cell activation and usually occurs immediately after CAR-T cell infusion. The main cytokines during cytokine storms are TNF and IFNg [34]. Therefore, with the CD47scFv-hinge-CD4TM-CD28-41BB-CD3*ζ* design, the CD47 CAR-T cell could reduce IFNg secretion.

In addition, in this study, we used the transmembrane domains of CD4 [35], and signaling through CD4 ensured T-cell activation and development, as well as stimulating T cells to recognize target antigens and prolong the interaction between T cells and antigens [36]. Finally, CAR-T cells were designed with two costimulation domains 4-1BB and CD28. The preclinical trial showed that CAR-T cells with two costimulation domains had better endurance, proliferation, and antitumor activity [37].

CD47 is involved in cancer cell metastasis and has a prominent role in endothelial migration [38]. The relationship between increased CD47 expression and tumor cell invasiveness and metastasis has also been clarified in previous studies. High expression of CD47 is found in nonsmall cell lung cancer [39], and these cancers could easily escape the immune system. The CD47 surface expression was remarkably increased after being adhesive to the endothelium. This phenomenon helps them to grow in the interstitial lung [40] and form metastases. CD47 modulates the actin in epithelial cells and participates in adhesing and migrating cells under the modulation of COX-2 protein [41]. Using specific anti-CD47 antibodies or siRNA reduced COX-2 expression compared to the control group [42]. microRNA-133a is able to target the CD47 protein that inhibits the proliferation, migration, and infiltration of laryngeal carcinoma cells [43]. CAR-T CD47scFv-hinge-CD4TM-CD28-41BB-CD3*ζ* cells in this study reduced the simultaneous expression of all three genes that have been shown to be involved in metastasis including beta actin, calreticulin, and COX-2. Thus, the CAR-T CD47 cells in this study, in addition to the effect of killing target cancer cells A549, also inhibited the expression of genes involved in metastasis. The possible explanation for this phenomenon is that the cells responsible for CD47-expressing metastasis were destroyed by CD47 CAR-T cells. Meanwhile, the remaining cells were cells that did not express CD47 and also expressed less of the beta actin, calreticulin, and COX-2 genes.

## 5. Conclusion

This is the first report on the generation of CD47scFv-hinge-CD4TM-CD28-41BB-CD3*ζ* CAR-T cells, demonstrating the effectiveness of CD47-CAR-T cells in against cancer cells A549. In addition, A549 cells, after exposure to CAR-T, decreased the expression of several genes that were involved in A549 cell adhesion and metastasis. Future studies in animal models are need for further testing the expression of beta actin, calreticulin, and COX-2 at the protein level. Solutions to optimize CD47-CAR-T cell therapy for different types of cancer should be warranted.

## Figures and Tables

**Table 1:** Primer use in qPCR.

Gene target	Primer name	Oligonucleotide sequence	PCR product size (bp)	Ref
CAR gene	TestCarF	5′-TTTTACCCCT CCGACATCGC-3′	253	
	TestCarR	5′-CAGCACGATCAGAGCCATCTTG-3′		

Human beta actin gene	actinF	5′-ATGAAGTGTGACGTGGACAT-3′	150	[21, 22]
	actinR	5′-CAGGAGGAGCAATGATCTTGAT-3′		

Cyclooxygenase 2 gene	COX2F	5′-CAAATGAGATTGTGGGAAAATTGCT-3′	300	[23]
	COX2R	5′-GATCATCTCTGCCTGAGTATCTT-3′		

Calreticulin gene	CALRF	5′-AGTTCCGGCAAGTTCTACGG-3′	260	[24]
	CALRR	5′-CCACAGATGTCGGACCAAA-3′		

Glyceraldehyde 3-phosphate dehydrogenase	GAPDHF	5′-TTGTCTCACTTGTTCTCT-3′	87	[25]
	GAPDHR	5′-ATGGGAGTTGTTTTCTTG-3′		

## Data Availability

The data used to support the findings of this study are available from the corresponding author upon request.
